# Factors Influencing Utilization of ASHA Services under NRHM in Relation to Maternal Health in Rural Lucknow

**DOI:** 10.4103/0970-0218.69272

**Published:** 2010-07

**Authors:** Manish K Singh, JV Singh, N Ahmad, Reema Kumari, A Khanna

**Affiliations:** Department of Community Medicine, Chhatrapati Shahuji Maharaj Medical University, Lucknow, U.P., India

**Keywords:** ANC, ASHA, early registration, NRHM

## Abstract

**Background:**

Under National Rural Health Mission (NRHM), ASHA (accredited social health activist) has been identified as an effective link to address the poor utilization of maternal and child health (MCH) services by rural pregnant women.

**Objective:**

To study the factors influencing utilization of ASHA services in relation to maternal health.

**Study Design:**

Cross-sectional.

**Setting:**

Primary Health Centre (PHC), Sarojininagar, Lucknow and its rural field area.

**Study Period:**

September 2007 to August 2008.

**Study Unit:**

RDW (recently delivered women) were considered as those who delivered a live newborn at PHC Sarojininagar, within a week of interview and belonged to villages within the confines of the PHC being served by ASHA.

**Materials and Methods:**

350 RDW were interviewed at their bedside, by a preformed and pretested schedule and then were followed-up after six weeks.

**Results:**

Utilization of ASHA services for early registration was significantly associated with age and religion of RDW. Young, educated and socio-economic class III RDW utilized ASHA services the maximum for early registration. Utilization of ASHA services for adequate ANC or antenatal care (100 iron and folic acid tablets, 2 tetanus toxoid injection and ≥3 antenatal visits) was also inversely associated with age of RDW. Young, Hindu, scheduled caste, middle school pass, Class III RDW and those with birth order one had high odds for utilization of ASHA services for adequate ANC. With regard to postnatal check-up, again young RDW with birth order one, Hindu RDW in reference to Muslim and RDW in socio-economic class III had higher likelihood for utilization of ASHA services. Caste-wise scheduled caste (SC) and other backward caste (OBC) RDW had higher odds for utilization of ASHA services. Educated RDW and those with educated husband had higher odds for utilization of ASHA services for postnatal check-up.

**Conclusion:**

Young, educated RDW with low parity, educated husband and belonging to higher socio-economic class had higher odds of utilization of ASHA services.

## Introduction

Global MMR is currently estimated to be 400 per lakh live births.(1) In South East Asia, India has a dubious distinction of having one of the highest MMR of 301 per lakh live births and Uttar Pradesh has a still higher MMR of 517 per lakh live births.(2) Most maternal deaths and pregnancy complications can be prevented by quality antenatal, natal and post-natal care. Current utilization of any antenatal care services in India is only 77% (72% in rural and 91% in urban areas).(3) Despite the efforts, utilization of MCH services by the rural community has not reached the desired level. Recently, efforts to address these issues have gained momentum with the formulation of National Rural Health Mission. Janani Suraksha Yojana (JSY), launched on 12^th^ April 2005, is a safe motherhood intervention under NRHM, with the objective of reducing maternal and neo-natal mortality by promoting institutional delivery among the poor pregnant women. JSY has identified ASHA as an effective link between the Government and the poor pregnant women. ASHA has been assigned the duty to identify beneficiaries and facilitate receipt of adequate antenatal, natal and postnatal care. The present study was initiated to assess the factors influencing utilization of ASHA services in relation to maternal health in rural Lucknow.

## Materials and Methods

The present study was carried out at Sarojininagar block PHC of Lucknow district, from 31^st^ August 2007 to 30^th^ August 2008. The objective of the study was to analyze the factors influencing the utilization of ASHA services in relation to maternal health by recently delivered women (RDW). RDW were considered as those who delivered a live newborn at PHC Sarojininagar within a week of interview and belonged to villages within the confines of the PHC being served by ASHA. The study design was observational cross-sectional.

As per NFHS-III,(3) utilization of any antenatal care services was 64% for rural Uttar Pradesh, whereas according to baseline facts- UP(4) the utilization of any antenatal care was found to be 68.5% for rural Lucknow. A sample size of 350 RDW was taken assuming a minimum of any antenatal care service utilization of 68% (*P*=68%) and applying the formula 4pq/d^2^.

Simple random sampling technique was followed to include the study unit in the study sample. Number of deliveries conducted monthwise, over the same period last year were enumerated. Following the trend of deliveries in the last year, expected number of delivery per month was observed to be on an average about ≥ 90. Therefore to get the desired sample size of 350 RDW within the stipulated time period, 36 RDW per month or 9 RDW per week were chosen randomly using lottery method from among the RDW at PHC Sarojininagar who were enlisted weekly.

The study tool consisted of a schedule that was prepared at the Department of Community Medicine, CSM Medical University, Lucknow in consultation with faculty members and guides after reviewing relevant literature on the topic. The schedule was tested initially on 10% of the sample size; the lacunae were discussed and corrected as per recommendations of guides.

The study was undertaken in two stages. In the first stage, RDW were interviewed at their bedside, regarding their biosocial characteristics, quality of antenatal and natal care received for the currently terminated pregnancy and the service facilitator. In the second stage, RDW were followed-up after six weeks of delivery to assess the postnatal care received after discharge and the facilitator. The data collected were analyzed and tabulated using SPSS-15. Multiple logistic regression analysis was used. For analysis purpose, adequate ANC was taken as receipt of ≥ 3 antenatal visits, 2 T.T injections and 100 I.F.A. tablets and receipt of postnatal care was taken as a minimum of one postnatal check-up within six weeks of delivery. ‘Others’ in the study included health care personnel excluding ASHA.

This study is a part of MD thesis and therefore suffers from constraints of resources and time. No caste division was studied among Muslims, as the numbers of Muslim RDW were very few.

## Results

Table 1 shows that out of the total 350 respondents majority 93.1% of RDW were Hindus whereas Muslims constituted 6.9% of the study sample. Among Hindus more than half belonged to scheduled caste and nearly one-third belonged to OBC.

**Table 1:** Age, caste and religion of respondents (*n*=350)

Religion	Age in years				Total
	20-24	25-29	30-34	35-39	
Hindu	85 (94.4)	211 (94.2)	28 (84.8)	2 (66.7)	326 (93.2)
	[26.1]	[64.72]	[8.59]	[0.61]	[100]
SC	50 (58.8)	108 (51.2)	12 (42.86)	0 (0.0)	170 (52.2)
	[29.4]	[63.5]	[7.1]	[0.0]	[100]
OBC	26 (30.6)	73 (34.6)	11 (39.3)	2 (100)	112 (34.4)
	[23.2]	[65.2]	[9.8]	[1.8]	[100]
General	9 (10.6)	30 (14.2)	5 (17.86)	0 (0.0)	44 (13.5)
	[20.5]	[68.2]	[11.4]	[0.0]	[100]
Muslim	5 (5.6)	13 (5.8)	5 (15.2)	1 (33.3)	24 (6.8)
	[20.8]	[54.2]	[20.8]	[4.2]	[100]

Total	90 (100)	224 (100)	33 (100)	3 (100)	350 (100)
	[25.7]	[64.0]	[9.4]	[0.9]	[100]

Values within ( ) parenthesis represent column percentage, and those within [ ] parenthesis represent row percentage.

Majority 64% of RDW were in the age group of 25-29 years, followed by 25.7% in the age group of 20-24 years. In both Hindus and Muslims, majority of RDW were in the age group of 25-29 years; 64.7 and 54.2% respectively.

Table 2 shows that in the study sample, majority of RDW 56.6% were illiterate and only 11.1% were high school educated and above. Caste-wise distribution among Hindus showed that majority of the SC RDW i.e. 65.9% were illiterate followed by 52.7 OBC RDW. About 40% general category RDW were illiterate. Higher percentage of Hindu RDW 11.7% had an educational qualification of high school and above, whereas it was 4.2% among Muslim RDW.

**Table 2:** Biosocial characteristics of recently delivered women in relation to their religion and caste (*n*=350)

Variables	Caste among Hindus			Religion		Total (*n*=350)
	SC (*n*=170)	OBC (*n*=112)	General (*n*=44)	Hindu (*n*=326)	Muslim (*n*=24)	
Educational status of RDW						
Illiterate	112 (65.9)	59 (52.7)	18 (40.9)	189 (58.0)	9 (37.5)	198 (56.6)
	[59.3]	[31.2]	[9.5]	[95.4]	[4.6]	[100]
Primary school	20 (11.8)	20 (17.9)	4 (9.1)	44 (13.5)	7 (29.2)	51 (14.6)
	[45.5]	[45.5]	[9.0]	[86.3]	[13.7]	[100]
Middle school	26 (15.3)	20 (17.9)	9 (20.5)	55 (16.9)	7 (29.2)	62 (17.7)
	[47.3]	[36.4]	[16.4]	[88.7]	[11.3]	[100]
High school	12 (7.1)	13 (11.6)	13 (29.6)	38 (11.7)	1 (4.2)	39 (11.1)
	[31.6]	[34.2]	[34.2]	[97.4]	[2.6]	[100]
Educational status of husband						
Illiterate	63 (37.1)	31 (27.7)	10 (22.7)	104 (31.9)	11 (45.8)	
	[60.6]	[29.8]	[9.6]	[90.4]	[9.6]	
Primary school	29 (17.1)	23 (20.5)	4 (9.1)	56 (17.2)	5 (20.8)	
	[51.8]	[41.1]	[7.1]	[91.8]	[8.20]	
Middle school	54 (31.8)	27 (24.1)	10 (22.7)	91 (28.0)	6 (25.0)	
	[59.3]	[29.7]	[11.0]	[93.8]	[6.2]	
High school	24 (14.1)	31 (27.7)	20 (45.4)	75 (23.0)	2 (8.3)	
	[32.0]	[41.3]	[26.7]	[97.4]	[2.6]	
Socio-economic class [Modified Pareek socio-economic class scale](5)						
III	4 (2.4)		14 (31.8)	25 (7.7)	0 (0.00)	
	[16.00]		[56.00]	[100]	[0.00]	
IV	92 (54.1)		28 (63.6)	192 (58.9)	15 (62.5)	
	[47.9]		[14.6]	[92.8]	[7.25]	
V	74 (43.5)		2 (4.5)	109 (33.4)	9 (37.5)	
	[67.9]		[1.8]	[92.4]	[7.63]	

Values within ( ) parenthesis represent column percentage, and those within [ ] parenthesis represent row percentage.

Majority of the husbands of RDW among both Hindus (31.9%) and Muslims (45.8%) were illiterate. Among general caste a maximum of 45.4% husband of RDW had an educational status of high school and above whereas among S.C. majority of RDW had illiterate husband 60.6%.

In the present study, none of the RDW as per modified Pareek socio-economic class scale(5) belonged to class I or II households. Majority of RDW 59.1% belonged to class IV households followed by class V. Religion-wise maximum number of both Hindu and Muslim RDW belonged to socio-economic class IV; 58.9 and 62.5%, respectively. Households belonging to general and OBC category were economically better off with lesser percentage of 4.5 and 29.5% of households respectively belonging to Class V in comparison to SC RDW 43.5%.

Table 3 shows that out of the 350 RDW studied, early registration (within 16 weeks) was reported by 249 RDW. On applying multiple logistic regression analysis to the sample of 249 RDW, utilization of services of ASHA for early registration was found to be significantly associated with age and religion of RDW. Inverse association was found for utilization of ASHA services for age, educational status and socio-economic class of RDW with respect to the reference. Young RDW, RDW belonging to socio-economic class V and illiterate RDW were more likely to avail ASHA services. Hindu RDW and SC RDW had higher odds of utilization of ASHA services.

**Table 3:** Multivariate logistic regression analysis of predictors influencing utilization of services of ASHA for early registration by RDW (*n*=249)

Predictors	β-coefficient	*P* value	Odds ratio (O.R)
Age in years			
20 to 24	− 18.63	0.00	8.10 E 09
24 to 29	− 18.17	0.00	1.28 E 08
30 to 34	− 17.47	0.00	2.58 E 08
34 to 39 (Ref**)			
Birth order of newborn			
One	0.60	0.65	1.83
Two	0.40	0.74	1.49
Three	0.36	0.76	1.43
Four	0.84	0.58	2.32
Five (Ref**)			
Religion			
Hindu	1.48	0.05	4.41
Muslim (Ref**)			
Caste			
S.C	0.03	0.97	1.03
O.B.C	− 0.44	0.42	0.64
General (Ref**)			
Educational status of RDW			
High school	− 1.13	0.21	0.32
Middle school	− 0.33	0.64	0.72
Primary school	− 0.03	0.97	0.97
Illiterate (Ref**)			
Educational status of husband
High school	0.32	0.72	1.38
Middle school	− 0.09	0.91	0.91
Primary school	0.22	0.79	1.24
Illiterate (Ref**)			
Socio-economic class			
III	− 0.75	0.55	0.47
IV	0.30	0.67	0.74
V (Ref**)			

(Ref**): Reference

Table 4 shows that out of the 350 RDW studied, adequate ANC (i.e. receipt of ≥ 3 antenatal visits, 2 T.T injections and 100 I.F.A) was received by only 34 RDW. On applying multiple logistic regression analysis on a sample of 34 RDW, to remove the confounding effects of other factors, none of the predictors were found to have a significant association with utilization of ASHA services for adequate ANC. Age of RDW was found to be inversely associated with utilization of ASHA services. RDW with birth order of newborn one, educational status of middle school pass followed by high school pass and belonging to class III had highest odds for utilization of ASHA services in respective category. RDW with high school pass husband were more likely to utilize ASHA services in reference to those with illiterate husband.

**Table 4:** Multivariate logistic regression analysis of predictors influencing utilization of services of ASHA for adequate ANC by RDW (*n*=34)

Predictors	β-coefficient	*P* value	Odds ratio
Age in years			
20 to 24	− 53.17	0.99	8.07 E 24
24 to 29	− 35.74	0.99	3.01 E 16
30 to 34 (Ref**)			
Birth order of newborn			
One	46.55	0.99	16.54 E 18
Two	14.63	1.00	2.24 E 06
Three (Ref**)			
Religion			
Hindu	− 15.25	1.00	2.39 E 07
Muslim (Ref**)
Caste
S.C	2.65		14.17
O.B.C	− 12.29	1.00	4.59 E 06
General (Ref**)			
Educational status of RDW
High school	12.80	1.00	3.62 E 05
Middle school	19.36		2.57 E 08
Primary school	3.47		32.05
Illiterate (Ref**)			
Educational status of husband			
High school	10.39		32656.86
Middle school	− 42.58	0.99	3.21 E 19
Primary school	− 29.34	0.99	1.80 E 13
Illiterate (Ref**)			
Socio-economic class			
III	1.82		6.17
IV	− 38.48	0.99	1.94 E 17
V (Ref**)			

(Ref**): Reference

Table 5 shows that out of the 350 RDW studied, 74 had a postnatal check-up (i.e. minimum of one postnatal check-up within 6 weeks of delivery). On applying multiple logistic regression to the sample of 74 RDW, none of the predictor was found to have a significant association with utilization of ASHA services for any postnatal check-up. Educational status of RDW was inversely associated with utilization of ASHA services. RDW in the age group of 25-29 years, with birth order one, Hindu RDW in reference to Muslim, and RDW in socio-economic class III had higher likelihood for utilization of ASHA services. Caste-wise SC and OBC RDW had higher odds for utilization of ASHA services. RDW with primary school pass husband had highest odds for utilization of ASHA services.

**Table 5:** Multivariate logistic regression analysis of predictors influencing utilization of services of ASHA for postnatal check-up by RDW (*n*=74)

Predictors	β-coefficient	*P* value	Odds ratio (O.R)
Age in years			
20 to 24	23.21	0.99	12.07 E 09
24 to 29	22.07	0.99	38.61 E 08
30 to 34 (Ref**)			
Birth order of newborn
One	49.29	0.99	25.50 E 20
Two	− 23.86	0.99	4.33 E 11
Three	− 3.70	1.00	0.03
Four	36.50	.	71.32 E 14
Five (Ref**)			
Religion			
Hindu	55.07	0.99.	82.74 E 22
Muslim (Ref**)			
Caste			
S.C	− 19.92	0.99	2.23 E 09
O.B.C	− 18.36	0.99	1.06 E 08
General (Ref**)			
Educational status of RDW			
High school	− 18.66	0.99	7.83 E 09
Middle school	− 1.12	1.00	0.33
Primary school	− 0.19	1.00	0.83
Illiterate (Ref**)			
Educational status of husband
High school	− 19.53	0.99	3.29 E 09
Middle school	− 19.33	0.99	4.01 E 09
Primary school	35.48	.	25.81 E 14
Illiterate (Ref**)			
Socio-economic class
III	63.67	.	44.68 E 26
IV	37.41	0.99	17.65 E 15
V (Ref**)		.	.

(Ref**): Reference

## Discussion

In the present study, educated RDW were found to be more likely to utilize ASHA services for early registration, adequate ANC and postnatal check-up. Previous studies(3,6) had also found educational level of women to have a positive influence on the utilization of antenatal services. As per NFHS III(3) detailed report also, the likelihood of a postnatal check-up increased with the educational level of the mother. Educated women being more aware and realizing the importance of early registration, adequate ANC and postnatal check-up are more likely to utilize the services of ASHA for the same.

In the present study, none of the RDW belonged to socioeconomic class I or II. Among the RDW, those belonging to socio-economic class III had the highest likelihood of utilizing ASHA services for early registration, adequate ANC and postnatal check-up as compared to those belonging to class IV and V. This is in accordance with the findings of previous studies(3,6) that also reported a sharp increase in the likelihood of receipt of antenatal and postnatal care, with the increase in the household’s wealth index. RDW belonging to class III were more likely to utilize the services of ASHA as they had lesser fear of losing a day’s daily wage compared to those belonging to class IV and V.

As per the present study, Hindus in reference to Muslims had higher odds for utilization of services of ASHA for early registration, adequate ANC and postnatal checkup. The finding agrees with that of NFHS-III, India.(3) Hindu RDW being more literate and economically well could be a contributory factor.

The present study revealed that among Hindus, scheduled caste RDW utilized the services of ASHA the maximum for early registration, adequate ANC and postnatal check-up. On the contrary, previous studies(6) had found that women from higher castes were more likely to avail antenatal and postnatal care. NFHS-III, India(3) also reported that antenatal care received was high among mothers who did not belong to SC/ST or OBC. The reason for discordance with the above study is better utilization of ASHA services by SC RDW, who were better convinced by ASHA.

As per the present study, young RDW had the highest likelihood of utilization of ASHA services for early registration, adequate ANC and postnatal check-up. The findings of the present study agree with earlier studies(3,7) that older women aged 35-49 years compared to younger women received much less antenatal care for their most recent birth. Utilization of antenatal services gradually declined with increasing age, this to some extent related to the greater anxiety, concern and receptive behavior among young RDW. However, no marked variation in utilization of postnatal check-ups has been reported by mother’s age in NFHS-III.(3)

In the present study, RDW with low parity had high odds for utilization of ASHA services. The findings are in accordance with previous studies(3,7) also that likelihood of receiving antenatal care declined sharply with rise in birth order. This can be partly attributed to greater home responsibilities of women with higher parity and non-availability of anyone to take care of their families in their absence.

## Conclusion

The present study lead us to the conclusion that educated RDW, those belonging to higher socio-economic class, Hindus in reference to Muslims, young RDW and those with low parity were more likely to utilize ASHA services for early registration, adequate ANC and postnatal check-up. On the other hand, contrary to previous studies, women from lower castes were more likely to avail antenatal and postnatal care. The reason for discordance is better approach of ASHA and her ability to connect and convince the women belonging to lower caste.

## Recommendation

Under NRHM, ASHA has been assigned the responsibility to counsel women regarding early registration, birth preparedness, adequate ANC, safe delivery, postnatal care etc and to mobilize the community and facilitate them in accessing these services for improvement in maternal health.

Counselling on antenatal care was found to be lagging (only 34 out of 350 i.e. 9.7% of the RDW received adequate antenatal care). So, extra efforts are needed to sensitize ASHA on these issues during training and by regular orientation programs.

In the present study, out of the 350 RDW studied, only 74 had at least one postnatal check-up. This can be attributed to the ignorance of majority of the women and their families regarding importance of postnatal care and also partly due to lack of effort on the part of ASHA, who also need to be sensitized on postnatal care. There is a need to enhance the knowledge and awareness of ASHA on the importance of postnatal care. She should be provided hands on training in postnatal care components by specialists. This will reflect into proper utilization of ASHA services for postnatal care.

Better and intense advocacy via IEC and BCC tools regarding the importance of complete antenatal care (2T.T, 100I.F.A, 3A.N.C check-up), institutional delivery, post-natal check-up as well as the facilitator role of ASHA for the same is needed in the community.
